# Complete chloroplast genome sequence of *Pueraria thomsonii*, an important traditional Chinese medicine plant

**DOI:** 10.1080/23802359.2019.1693301

**Published:** 2019-11-22

**Authors:** Xiao-Rong Miao, Jun-Qi Niu, Ai-Qin Wang, Dao-Bo Wang, Jing Fan

**Affiliations:** a College of Biology and Pharmacy, Yulin Normal University, Yulin, China;; b Guangxi Key Laboratory of Agricultural Resources Chemistry and Biotechnology, Yulin, China;; c College of Life Sciences, Leshan Normal University, Leshan, China

**Keywords:** *Pueraria thomsonii*, complete chloroplast genome, legume, illumina sequencing

## Abstract

*Pueraria thomsonii* is a leguminous plant with high root yield and starch content. It is also a medicinal material in the Chinese pharmacopeia. However, the raw materials of *P*. *thomsonii* are often confused with some non-medicinal Pueraria plants. To enrich the genetic resources of *P*. *thomsonii* and guide its molecular identification, the complete chloroplast genome was sequenced and reported. The total genome of *P*. *thomsonii* is 153,434 bp in length. consisting of two inverted repeat regions (IR_S_, 25,640 bp each) separated by a large single-copy (LSC, 84,155 bp) and a small single-copy region (SSC, 17,999 bp). The overall GC content is 35.41%. It contains 130 genes, including 85 protein coding genes, 8 rRNA genes and 37 tRNA genes. Phylogenetic analysis showed that *P*. *thomsonii* could be distinguished from other plants and closely related to the legume *Pachyrhizus erosus*. This study enriches the genetic information of *P*. *thomsonii* and contributes to the screening of excellent germplasm.

Pueraria plant is a perennial vine of the papilionaceae family. It is reported that there are nearly 20 species in the world (Egan et al. 2016), among which there are 9 species and 2 variants of Pueraria in China, but only the *Pueraria lobata* (Willd.) Ohwi. and *Pueraria thomsonii* Benth were used as medicinal materials and archived in the Chinese pharmacopeia (Zhao et al. 2011; Wang et al. 2018). Due to the scarcity of wild *P. lobata* resources, *P*. *thomsonii* can also produce isoflavones and is used to prevent various chronic diseases, it is widely grown to supply medicinal materials (Wong et al. 2015; Liang et al. 2017). However, non-medicinal plants of the genus Pueraria often appear in the market, therefore, the accurate identification of *P*. *thomsonii* can guarantee the source and quality of pueraria. Chloroplast genome has been widely used in plant evolution and taxonomy due to its maternal inheritance and conserved structure (Fan and Huang 2019). However, the complete chloroplast genome of *P*. *thomsonii* has not been reported. In this work, the chloroplast genome sequence of *P*. *thomsonii* was decrypted for the first time, which can better understand the genetic background of Pueraria and provide a basis for species identification of *P*. *thomsonii*.

The leaves of *P*. *thomsonii* were collected from the medicinal botanical garden of Guangxi university in Nanning, Guangxi (108°33′45″E, 22°82′13″N), the specimen (YS20171014) was stored in the herbarium of Yulin normal university. Total DNA of *P*. *thomsonii* was extracted from fresh leaves by SDS method, and sequenced by Illumina HiSeqXten platform. The obtained genome sequences were de novo assembled using SPAdesv.3.11.0 (Bankevich et al. 2012), low-quality reads and adapters were removed by FastQC software (Andrews 2010), and finally annotated by Plann software (Huang and Cronk 2015). The chloroplast genome size of *P*. *thomsonii* (GenBank accession no. MN515038) is 153,434 bp and has four sub-regions: a large single copy (LSC) of 84,155 bp and a small single copy region (SSC) of 17,999 bp, which are separated by two inverted repeats (IRs) of 25,640 bp. It contains 130 genes, including 85 protein-coding genes, 37 tRNA genes and 8 rRNAs, 18 genes (7 Protein coding genes, 4 rRNAs, and 7 tRNAs) are duplicated in the inverted repeat regions, the total GC content is 35.41%.

To analyze the phylogeny of *P*. *thomsonii*, 39 complete chloroplast genomes were aligned using MAFFT7.037 software (Katoh and Standley 2013), then the Maximum Likelihood (ML) phylogenetic tree was constructed by Mega-X v10.0.5 software (Kumar et al. 2018), with the operating parameters of GTR + G model and 1000 bootstrap replicates. The result of ML phylogenetic tree showed that *P*. *thomsonii* is closely related to *Pachyrhizus erosus* (Figure 1). Our results provide useful resources for molecular identification and phylogeny of *P*. *thomsonii*.

**Figure 1.**
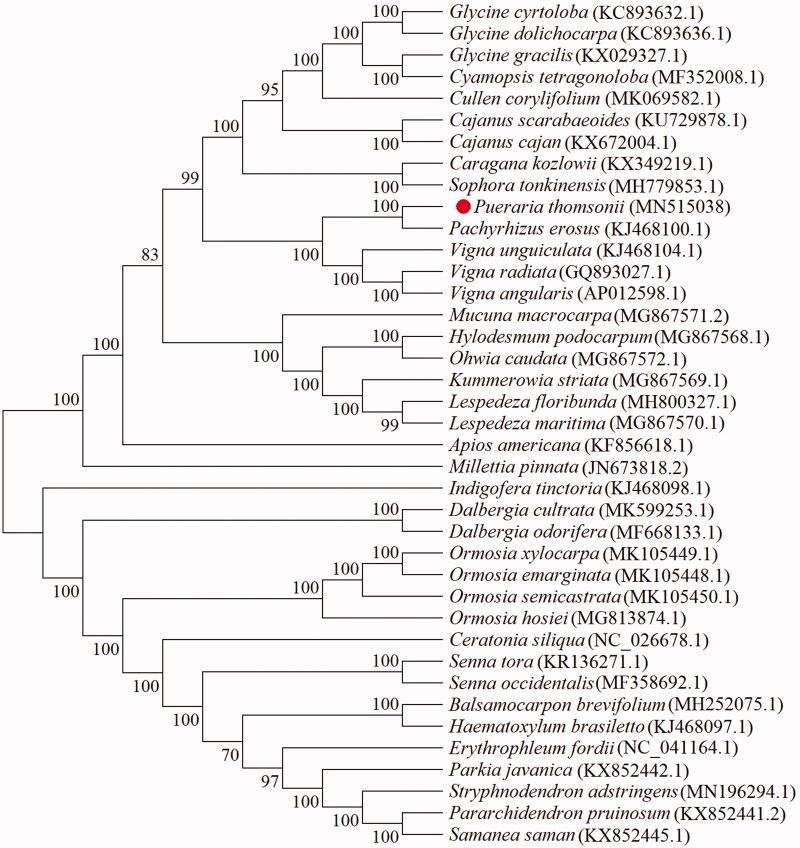
The Maximum Likelihood (ML) phylogenetic tree based on the 39 plant chloroplast genome. Note: The number near each node represents the support value of 1000 bootstrap replicates.
